# Digital Compression in Aneurism

**Published:** 1861-03

**Authors:** 


DIGITAL COMPRESSION IN ANEURISM. —In a recent number of the *Gazette Hebdomadaire*, we find the statement that M. Bouvier communicated to the Paris Society of Surgery two cases of traumatic aneurism, cured by digital compression, by M. Mirault, of Angers. The first case was that of a man, twenty-three years of age, who perceived a small tumor gradually develop itself at the bend of the elbow, after the operation of phlebotomy. The brachial artery was compressed, and a cure was effected in thirty-one hours. The second case occurred in a child, nine years old, in whom the origin of the temporal artery had been wounded by the bill of a cock, giving rise to a tumor about the size of a crab apple. As it was impossible to control the artery between the heart and the tumor, direct compression was made on the swelling at different times, and it was discontinued during the night. A cure resulted in eighty-five hours.

